# The Influence of Contour Form Geometric Features and the Number of Cutting Passes on the Surface Quality Characteristics and Critical Points of Cutting Tools Fabricated by Wire Electrical Discharge Machining (WEDM)

**DOI:** 10.3390/mi16020227

**Published:** 2025-02-17

**Authors:** Amir Alinaghizadeh, Bahman Azarhoushang, Mohammadjafar Hadad

**Affiliations:** 1 KSF—Institute for Advanced Manufacturing, Furtwangen University, 78532 Tuttlingen, Germany; 2 Energy and Sustainable Development Research Center, Semnan Branch, Islamic Azad University, Semnan P.O. Box 35145-179, Iran; 3 School of Mechanical Engineering, College of Engineering, University of Tehran, Tehran 1417466191, Iran; mjhadad@ut.ac.ir

**Keywords:** electric discharge, wire cut, form-cutting tool, recast layer, cutting passes

## Abstract

Since one of the effective methods for producing the form-cutting tools used in the form-turning process involves utilizing a wire cut machine, the effect of the geometric characteristics of the form contour on reducing the negative effects of the recast layer was investigated in this research. The basic assumption of the components for each type of profile form is based on a combination of four modes, i.e., concave arc, convex arc, flat surface, and oblique surface. Based on this, samples were fabricated as cutting tools with three different radii: a convex arc, a concave arc, and a flat surface. During the wire electrical discharge machining (WEDM) operation, one-pass mode was used to create a rough surface, two passes resulted in a semi-finished surface, and three passes resulted in a finished surface. Furthermore, the difference between the surface quality of the recast layer in the two areas above the workpiece or the wire entry point and the bottom area of the workpiece or the wire exit point was studied. Finally, the effect of the direction, size of the curvature and the number of passes in the electric discharge process of the wire on the recast layer was shown, and it was observed that with the increase in the number of passes in WEDM, the thickness of the recast layer was reduced, along with the uniformity of the cutting contour section in the areas close to the cutting region. The entry of the wire was greater than that in the areas near the exit of the wire.

## 1. Introduction

Advancements in the constituent materials of components and their geometric complexity have led to fundamental changes in machining methods. Currently, there is a growing demand for materials with outstanding properties, such as wear resistance, the ability to function at high temperatures, light weight, corrosion resistance, etc., [1,2,3,4]. Moreover, traditional machining cannot satisfactorily produce complex geometries and shapes, necessitating the use of advanced machines capable of simultaneous movement in multiple axes [5,6]. Additionally, the integration of various machining processes into a single machine has seen tremendous growth in recent years [7,8]. One of the conventional and reliable methods for producing specific forms in the machining process is the use of “form-cutting tools”. Although the cutting forces are high due to the high contact surface between this type of tool and the workpiece, this method has always been a suitable alternative for the milling process, offering point-to-point interpolation through machine controller while using conventional point contact cutting tools. To produce form-cutting tools, three methods are used. When a large number of tools are required, it is recommended to make the tools out of tungsten carbide materials, and the best method for producing them is the powder metallurgy (PM) method. In this process, the desired cutting tools can be produced repeatedly. When the number of form-cutting tools required is lower, the grinding method is recommended. In fact, this method involves making a raw insert using grinding machines [9,10]. Ground inserts are utilized in two ways to create a profile form. In the first mode, standard grinding wheels are used in conventional grinding machines for processes such as flat grinding, surface grinding, and cup grinding, are used to machine the form area. Each contour form area is formed through one of the grinding wheels according to its geometry, and ultimately, using multiple grinding wheels, the final shape of the form-cutting tool is created. Another method used to create form-cutting tools is the “profile-grinding” method [11,12]. In this method, reverse profiling is used to create the desired form profile on the grinding wheel, and then the profile is transferred to the cutting tool through the grinding process. The key criterion for employing this method is the number of form-cutting tools. Besides influencing a significant portion of the grinding wheel’s diameter, other costs such as tool wear and the cost of stopping operations to repair the formed profile on the grinding wheel occur during the grinding process when this method is used [13,14]. Grinding form-cutting tools made of materials with very high mechanical strength, such as cubic boron nitride, incurs high costs. Additionally, profiling on super-abrasive grinding wheels involves many challenges, and moreover, form tools usually have low production volumes and high form profile variations. Therefore, in some cases, for economic reasons, this method is not considered a suitable choice [15,16,17]. The third method for producing form-cutting tools, suitable for single-piece production and prototyping, is the use of the wire electrical discharge machining (WEDM) process. This process enables the production of form-cutting tools with considerable speed and accuracy and is capable of integration with output programs from CAM (Computer-Aided Manufacturing) software. The form-cutting tools produced by wire EDM machines or grinding machines must have desirable process outputs, such as proper surface quality, correct geometry, and cutting edge sharpness, to be approved by the customer. To achieve this goal and achieve high tolerances and a good surface finish on the desired surface, the grinding process is usually used as the final process due to the high speed and efficiency of this method. Although the WEDM method is sometimes used as a competitor, or even the only method, it still cannot be considered a complete substitute for the grinding process. For form-cutting tools with replaceable cutting edges, which consist of a tool holder and a cutting insert made of cubic boron nitride (CBN) and polycrystalline diamond (PCD), the WEDM process is a suitable choice. Wire EDM machines are considered a suitable and scalable choice. This method is ideal for producing 5 to 20 pieces of form-cutting tools; this was determined based on the complexity of the initial machining stages and the accuracy of the initial adjustments in form machining. However, economically, the use of the wire EDM method is not recommended for producing large batches of products [18,19,20]. Form-cutting tools come into contact with a wide range of workpieces and may experience phenomena such as vibration and chatter due to the presence of resonance and noise. Low cutting speeds and high feed rates are among the factors that can cause this problem. The use of form-cutting tools is considered a general solution to increase accuracy and reliability, which has significantly improved their machining capability and performance accuracy in creating unique profiles over time. These tools offer high performance guarantees and demonstrate numerous examples of their application across various industries, resulting in outstanding benefits such as reduced production costs per workpiece, elimination of tool adjustments after each change, increased production efficiency, and significant time savings through the elimination of tool changes. The cutting edge in form-cutting tools has the inverse shape of the workpiece, and the correct depth can be achieved in a single pass [21,22]. Horizontal, forward, or backward movements are created using the machine’s motion axes. In the form turning operation, the feed motion applied to the form cutting tools can be executed on workpieces with varying diameters. Typically, when machining is performed using form-cutting tools, a significant portion of the workpiece is produced. In the form machining process, the chip formation is not uniform, and in some areas, chips are accumulated or fragmented based on the desired form profile. Manufacturing form-cutting using wire electrical discharge machining (WEDM) offers comprehensive advantages despite also being associated with specific limitations [23]. These advantages include the capability to create unique contours on the cutting edge and the ability to use standard cutting inserts and modify their cutting-edge shape to achieve the desired form. This method is suitable for low-volume production of cutting tools, as well as for research projects and concept-based products. Additionally, it eliminates the need to use molds and powder metallurgy equipment to produce specialized cutting inserts. WEDM also enables the creation of narrow grooves and sharp angles on the cutting edge. Furthermore, it enhances the flexibility that can be achieved while producing cutting angles on the tool edge by integrating CAD/CAM system capabilities with three- and five-axis wire EDM machines, even utilizing rotational axes. The primary limitation of WEDM lies in the relatively weak surface characteristics achieved using this method. The formation of a solidified layer is typically observable due to the melting and re-solidification of workpiece materials on the machined surface [24,25]. In rough machining, more thermal energy is transferred to the workpiece material, resulting in an increase in material melting. Some of the molten materials are removed due to the pressure wave created in the absence of plasma channels, while others form a thick solidified layer on the machined surface due to re-solidification by pressure waves. As rough cutting involves significantly longer pulse durations, it intensifies sparking. Consequently, a high density of spherical micron-sized particles, along with fine pores, indentations, and molten debris, is observed on the surface. Upon solidification of the molten metal, large craters are formed on the machined surface, indicating a high surface roughness. Conversely, in finish cutting, very fine electrical discharges are generated, melting only a small amount of material on the machined surface, which is easily removed by the pressure wave [26,27,28]. SEM confirms the entrapment of some air bubbles, which are released as the remaining molten material solidifies, creating small holes on the machined surface. In contrast, the tendency to form micron-sized particles or spherical particles, apart from a few small craters and molten particles, significantly decreases in the skim-cutting mode. The very fine discharge pulses, characterized by very low energy density, only melt a small amount of material on the machined surface, which is easily removed by the pressure wave, ultimately creating micro-/nano-holes on the machined surface [29,30,31]. The present study investigates the effect of geometric profile features in order to mitigate the adverse effects of the recast layer. The main assumption is based on creating various profile forms utilizing fundamental geometric elements, namely concave curvature, convex curvature, a flat surface (perpendicular region), and an inclined surface. Accordingly, experimental samples of high-speed steel (HSS) were made into form-cutting tools with three different radii of convex curvature, three different radii of concave curvature, and a flat surface, resulting in a total of seven different configurations. During the wire electrical discharge machining (WEDM) process, three machining conditions were utilized: rough cutting (with one wire cut pass), semi-finish cutting (with two wire cut passes), and finish cutting (with three wire cut passes). Thus, the total number of experimental samples was 21. Additionally, the difference in the quality of the recast layer was studied in two areas: the upper part of the workpiece or the wire entry point and the lower part of the workpiece or the wire exit point. Finally, the influence of curvature direction, curvature magnitude, and the number of passes in the wire electrical discharge machining process on the recast layer was demonstrated. Research on advanced machining methods has focused on overcoming the limitations of traditional approaches with regard to handling complex geometries and high-performance materials. Studies have shown that while grinding ensures high precision, challenges such as tool wear and economic constraints limit its applicability in certain cases [9,10]. WEDM has emerged as a flexible alternative, particularly for low-volume and prototype production, yet concerns about the surface integrity being affected by the recast layer remain [13,14]. Prior works emphasize the need to optimize machining parameters to minimize these effects. This study builds on these insights by investigating the impact of geometric profile features on recast layer formation, aiming to refine WEDM for improved tool performance.

## 2. Materials and Methods

**Figure 1:**
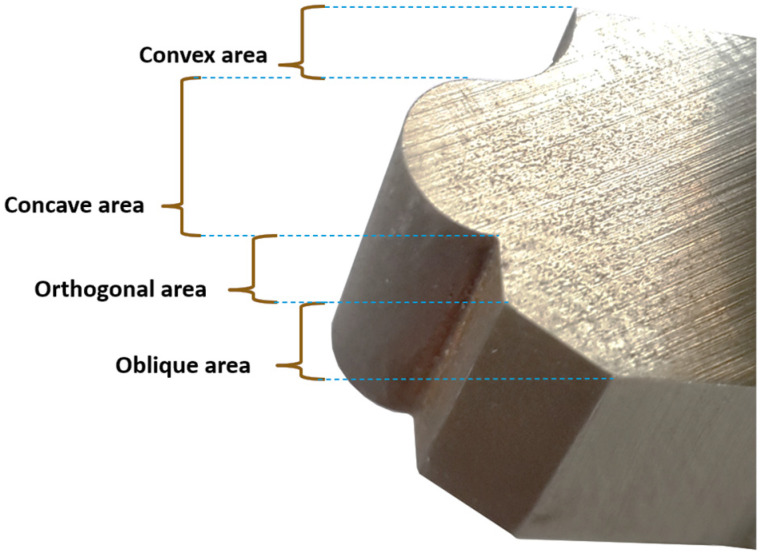
An example of a form-cutting tool, resulting from the combination of different geometric components.

The cutting-edge profile in form-cutting tools used in the form machining process is composed of a combination of four geometric components, namely convex, concave, flat smooth, and sloped flat surfaces. Figure 1 illustrates an example of a form profile formed by a combination of all four mentioned geometric components in a form-cutting tool. As depicted in the figure, it is evident that each desired form profile, through the combination of different types and sizes of geometric components, can be extended and presented. The width and angle of the convex, concave, flat smooth, and sloped flat regions are as follows: convex, with a radius of 2 mm and an angle of 90°; concave, with a radius of 2 mm and an angle of 180°; the flat smooth or orthogonal area, with a width of 2 mm; and the sloped flat or oblique area, with a width of 2 mm and an entry angle of 45°.

In order to investigate the effect of different form profile geometries on the qualitative surface properties with a recast layer, high-speed steel (HSS) was utilized as the cutting tool material. The chemical composition and some of the standard designations for HSS are presented in Table 1.

**Figure 2:**
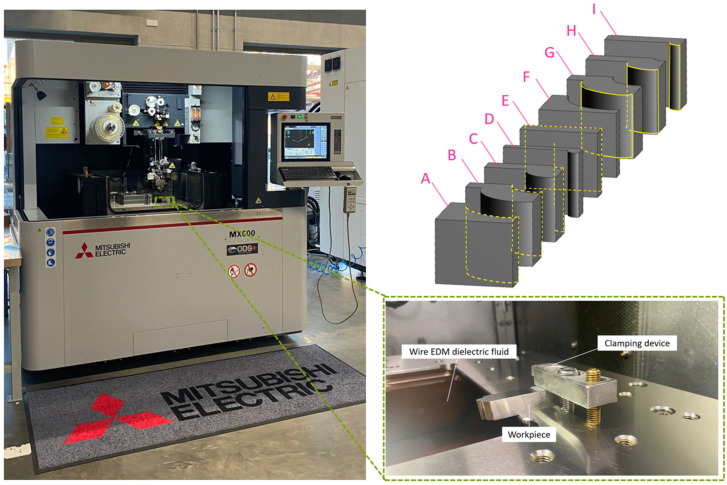
The machine used in the WEDM process and the seven contours considered to represent the cutting edge of the tool (named from A to I) and process parameters for one-, two-, and three-pass strategies.

In this study, a Mitsubishi MX600 wire electrical discharge machining (WEDM) machine, manufactured by Mitsubishi Electric in Japan, was employed, accompanied by seven different contour geometries as cutting-edge form tools, as depicted in Figure 2. Consistent with previous studies, a closed-loop constant-tension control system was utilized to enhance stability in wire electrode tension during machining. In this system, the wire electrode tension was set to a predetermined value of 5 N. Additionally, apart from minor fluctuations of approximately 0.5 N which occurred during reversals, the wire tension remained uniform and constant to meet the tension requirements in the machining process.

ToolBrass—Wire 0.25 mmWorkpieceHSS block (as a cutting tool for machining of VCN 150 with a diameter of 12 mm)ParameterSpecification of spark energy (1 pass):Pass No. 1: IP:14, ON time: 0.75 μs, OFF time: 30 μs, V_G_: 80 v, WT: 1 N/m, WS: 8.4 m/min, DF:8 (L/m), FA: 10 mm/min, H: 0.156 mmSpecification of spark energy (2 passes):Pass No. 1: IP: 14, ON time: 0.75 μs, OFF time: 30 μs, V_G_: 80 v, WT: 1 N/m, WS: 8.4 m/min, DF:8 (L/m), FA: 10 mm/min, H: 0.186 mmPass No. 2: IP: 9, ON time: 0.75 μs, OFF time: 30 μs, V_G_: 20 v, WT: 1.4 N/m, WS: 9 m/min, DF:0 (L/m), FA: 2.775 mm/min, H: 0.140 mmSpecification of spark energy (3 passes):Pass No. 1: IP:14, ON time: 0.75 μs, OFF time: 30 μs, V_G_: 80 v, WT: 1 N/m, WS: 8.4 m/min, DF:8 (L/m), FA: 10 mm/min, H: 0.186 mmPass No. 2: IP:9, ON time: 0.75 μs, OFF time: 30 μs, V_G_: 20 v, WT: 1.4 N/m, WS: 9 m/min, DF:0 (L/m), FA: 2.775 mm/min, H: 0.140 mmPass No. 3: IP:4, ON time: 0.45 μs, OFF time: 30 μs, V_G_: 14 v, WT: 1.4 N/m, WS: 6.8 m/min, DF:0 (L/m), FA: 2 mm/min, H: 0.128 mmDielectricOil—oil bathIP: Intensity of power (base of the Mitsubishi table); ON time: pulse time during discharging; OFF: pulse interval time; VG: gap voltage during discharging; WT: wire tension; WS: wire traveling speed; DF: dielectric fluid flow rate (L/m); FA: actual feed rate of electrode in cutting direction; and H: distance between electrode center and workpiece surface.

**Figure 3:**
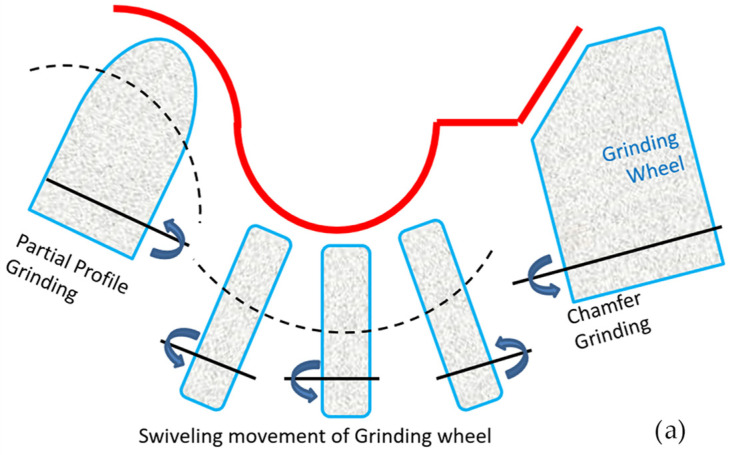

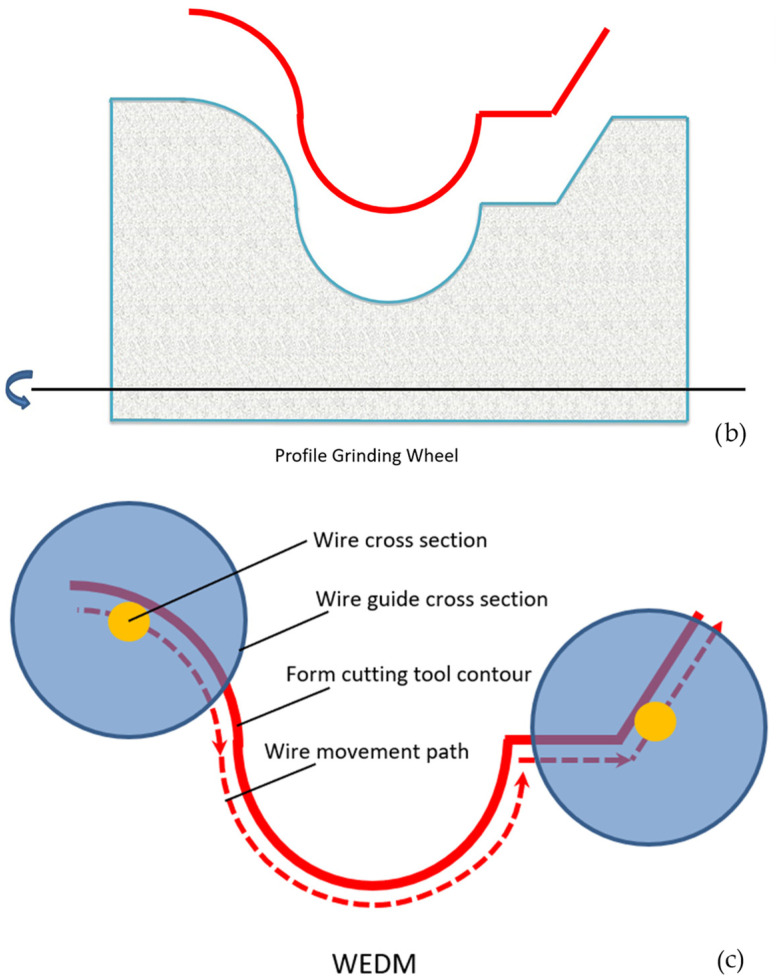
Common methods (**a**) Grinding, (**b**) profile grinding and (**c**) electric discharge by wire to produce the form-cutting tool shown in Figure 1.

Various methods are employed for the fabrication of form-cutting tools. Figure 3 illustrates the types of methods used. The final characteristics of the cutting tools are ultimately measured based on the fabrication method. According to previous studies, considering the presence of the recast layer and its influence on the cutting surface, the number of passes in the WEDM process was varied to compare three conditions: rough (utilizing a single wire-cut pass), semi-finish (employing two wire-cut passes), and finish (using three wire-cut passes). With regard to the explanations provided, the selected samples were characterized geometrically at seven levels and, in terms of WEDM process parameters, they underwent three levels of variations, resulting in a total of 21 samples examined in this study. The seven types of geometries include convex curvature with small (curvature radius: 2 mm), medium (curvature radius: 5 mm), and large (curvature radius: 8 mm) radii, a flat surface, and concave curvature with small (curvature radius: 2 mm), medium (curvature radius: 5 mm), and large (curvature radius: 8 mm) radii.

**Figure 4:**
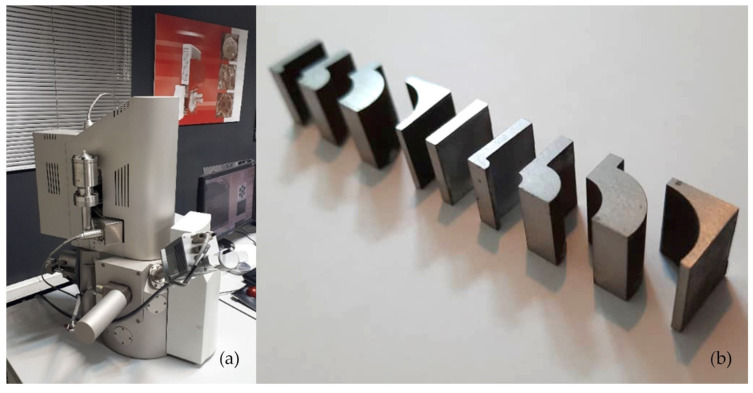
(**a**) SEM microscope used with features specification and (**b**) Test samples with different curvatures in terms of direction (convexity and concavity) and size.

**Figure 5:**
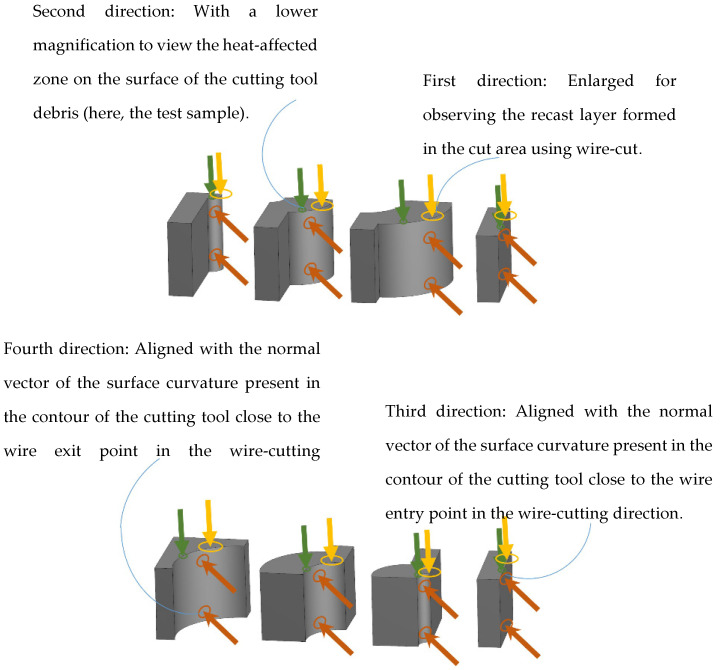
Selected directions for SEM imaging.

In Figure 4, the effect of the recast layer was examined using scanning electron microscopy (SEM). A Tescan SEM, manufactured in Brno, Czech Republic, was utilized for this analysis. Samples cut via WEDM were photographed from various orientations, and the results were compared in terms of surface characteristics. Figure 4 illustrates the samples, while Figure 5 shows the selected orientations used for SEM imaging.


**Feature**

**Specification**
TypeField-Emission Scanning Electron Microscope (FE-SEM)Electron SourceSchottky Field EmissionX-ray DetectorSingle Silicon Drift Detector (SDD)

**Figure 6:**
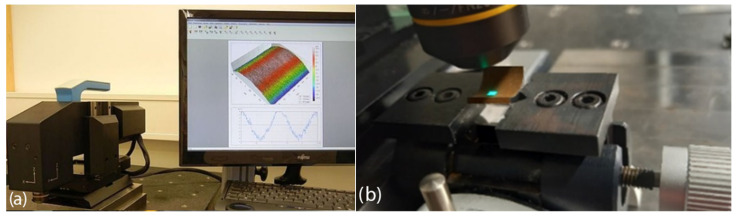
(**a**) Three-dimensional measurement of the sample surface by confocal scanning method and (**b**) Measurement range on the test sample with concave curvature.

To examine the surface properties of the recast layer, a confocal microscope was utilized and adjusted to ensure alignment between the image registration direction and the normal vector of the recast layer surface. A NanoFocus confocal microscope, manufactured in Oberhausen, Germany, was used for this analysis. Figure 6a depicts the microscope used to examine the surface of various samples in this study.


**Parameter**

**Specification**
Microscope Model and Typeµsurf Confocal Microscope (Portable)Lens and Focal Distance50 µm balance lens; 0.5 mm focal distanceMeasurement Range and Imaging Mode1.6 × 1.6 mm (without stitching); depth-up acquisition

Figure 6b illustrates the area designated for image registration. Due to the limited number of samples, the middle region of the samples’ height was selected to ensure equal distances between the entry and exit points of the wire. Given this method’s advantages when it comes to generating three-dimensional images, various surface features—such as surface roughness, texture variations, height profiles, and localized deformation patterns—were measured and compared among the samples.

## 3. Results and Discussion

**Figure 7:**
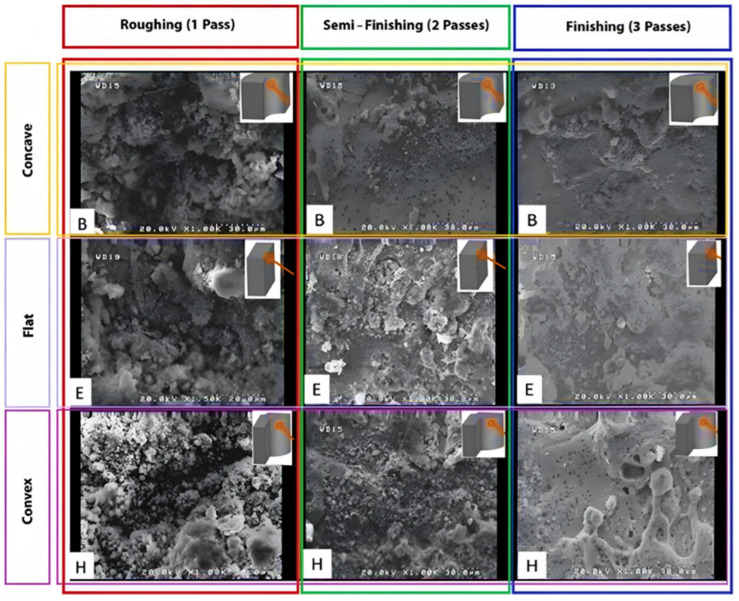
SEM images of cutting tools of various forms (samples B, E and H, according to the nomenclature of Figure 2), based on the geometric characteristics and the number of different cutting passes.

SEM observations revealed that single-pass machining resulted in a high density of molten particles, pits, and fine cavities on the machined surface. Figure 7 displays the surface morphology of the machined tools with different geometries obtained through one, two, and three cutting passes via the WEDM process. Indeed, a comparison was made among three different sizes of curvature radii for concave and convex states for rough, semi-finish, and finish cuts.

The recast layer is formed due to the melting and subsequent re-solidification of materials. In particular, a noticeable amount of this layer is observed in rough cuts (single pass) when comparing machined samples with one, two, and three cutting passes. The reason for this observation lies in the fact that, for rough cuts, the electrical discharge possesses a high energy density per unit time, increasing the spark intensity and melting more material, consequently forming larger cavities on the machined surface. Some of the molten material is washed away by the dielectric fluid under pressure, while some gas bubbles are trapped within it. As the remaining molten material solidifies again, these gas bubbles collapse, creating fine cavities on the machined surface. In the case of three-pass cuts (finish cuts), regardless of the sample geometry being smooth or having concave and convex curvatures, the ultra-fine discharge pulses melt only a small amount of material, which can be easily washed away by the separated particles. The absence of electrical discharge channels in the dielectric fluid facilitates this process. Consequently, the significant formation of the recast layer is reduced in the finish cutting mode.

Another significant point is the effect of wire engagement length with convex, flat, and concave surfaces, which increases from low to high. This aspect is illustrated in Figure 7 for convex and concave samples with different radii of curvature. With a longer engagement length, each point on the surface is exposed to electrical discharge for a longer period, resulting in a higher temperature. Consequently, it becomes more susceptible to heat damage during cooling, which may be caused by the dielectric fluid. Moreover, a longer period of exposure to heat causes the melted materials at the cutting position to “round up”, and with this pattern, the solidified particles and deposits are scattered on the surface. This phenomenon has been described in other studies as “spherical debris” and “irregular debris”. In all of the SEM images shown in Figure 7, the spherical nature of the visible components on the surface decreases from sample C to sample A, and the particle size decreases from the right side, which corresponds to concave samples, to the left side, which corresponds to convex samples. This is because, on the left side, during the electrical discharge process, two divergent convex surfaces come into contact with each other: one is related to the wire, and the other is related to the sample geometry. Therefore, there will be less engagement length for convex samples, meaning less exposure time to heat and, consequently, finer surface features. These differences in surface characteristics can be observed for all of the electrical discharge conditions of the samples.

**Figure 8:**
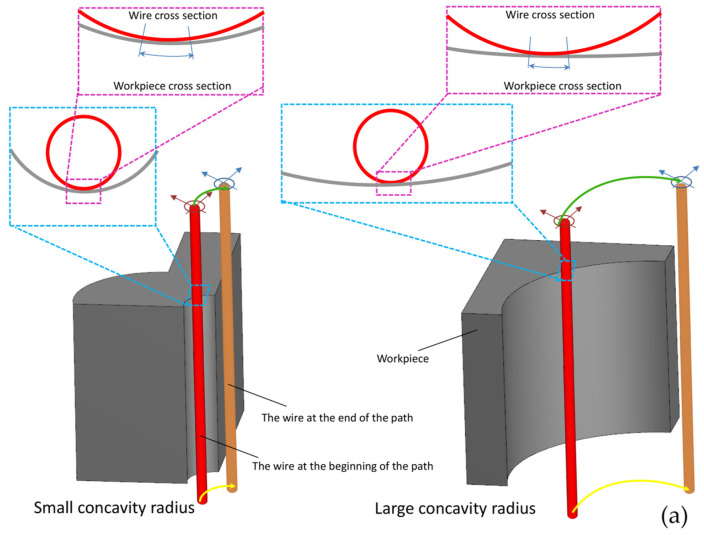

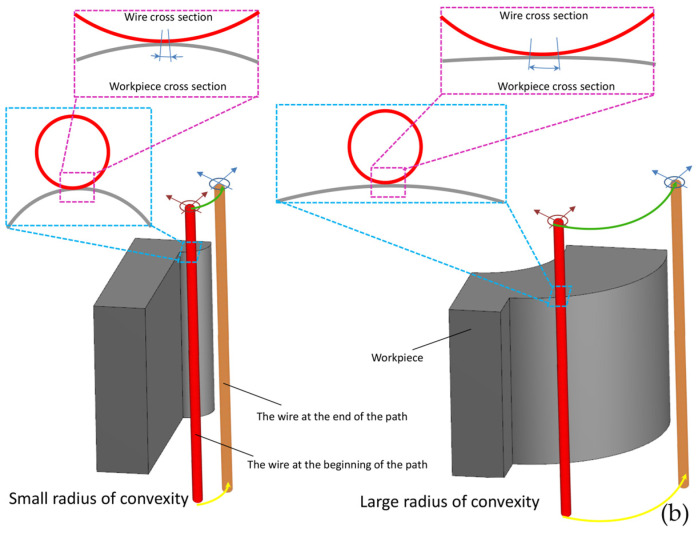
The length of the contact segment between the wire and the surface of the workpiece for samples with (**a**) convex and (**b**) concave surfaces with large and small radii [33].

Another notable point is the influence of wire engagement length with convex, flat, and concave surfaces, which increases from low to high, respectively. This aspect is illustrated in Figure 8 for convex and concave samples with different radii of curvature. With a longer engagement length, each point on the surface is exposed to electrical discharge for a longer period, resulting in a higher temperature. Consequently, it becomes more susceptible to heat damage when cooled by the dielectric fluid. Moreover, a longer period of exposure to heat causes the melted materials at the cutting position to “round up”, and with this pattern, the solidified particles and deposits are scattered on the surface.

In the SEM images shown in Figure 7, the spherical nature of the visible components on the surface decreases from samples C to samples A, and the particle size decreases from the top row, which corresponds to concave samples, to the bottom row, which corresponds to convex samples. This is because, on the bottom row, during the electrical discharge process, two divergent convex surfaces come into contact with each other: one is related to the wire, and the other is related to the sample geometry. Therefore, the engagement length will be shorter for convex samples, meaning a shorter period of exposure to heat and, consequently, finer surface features. These differences in surface characteristics can be observed for all of the electrical discharge conditions of the samples.

**Figure 9:**
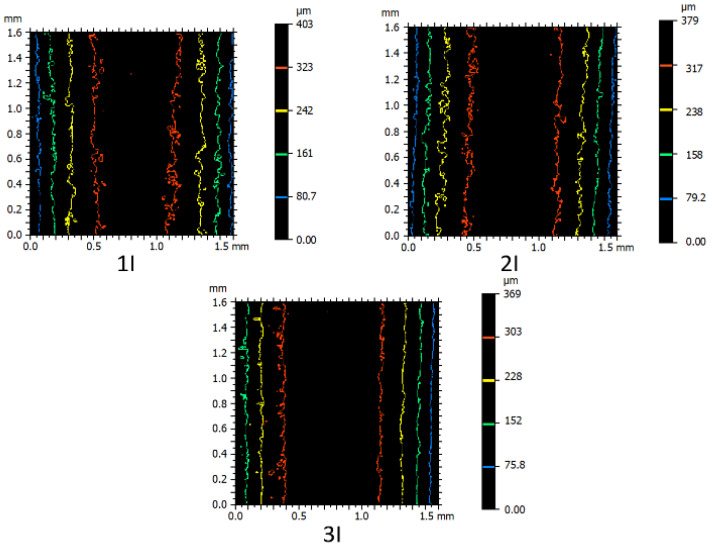
Contour color spectrometry to compare the cut samples with a small radius convex surface through the process of electric discharge with wire in three states: rough, semi-finished, and finished (Sample I according to the naming of Figure 2).

Another significant point is the impact of wire engagement length with convex, flat, and concave surfaces, which increases progressively. This aspect is depicted in Figure 9 for convex and concave samples with varying radii of curvature. With a longer engagement length, each point on the surface is subjected to electrical discharge for an extended duration, resulting in higher temperatures. Consequently, during cooling by the dielectric fluid, there is a greater susceptibility to heat damage. Additionally, prolonged exposure to heat causes the melted materials at the cutting position to assume a “rounded” shape, dispersing solidified particles and deposits across the surface.

In the SEM images presented in Figure 7, the spherical nature of visible components on the surface decreases from samples C to samples A, and the particle size diminishes from the top row, corresponding to concave samples, to the bottom row, which represents convex samples. This is because, in the bottom row, during the electrical discharge process, two divergent convex surfaces come into contact with each other: one is related to the wire, and the other is related to the sample geometry. Therefore, there will be less engagement length for convex samples, implying a shorter period of exposure to heat and, consequently, finer surface features. These differences in surface characteristics are observable across all electrical discharge conditions of the samples.

Furthermore, the contour color spectroscopy method, as illustrated in Figure 9, can serve as a suitable basis for comparing the recast layer among the patterned samples. According to the figure, this comparison was conducted for samples fabricated with one, two, and three passes. Regarding the geometric features, the smallest curvature radius (concave—small radius: piece I) was selected for contouring. The color of each contour indicates a specific height relative to the reference point, while the distance between contours of different colors represents the extent of height variation. Changes in directions and deviations in other directions also indicate the development of features of uniform height points on the surface. Additionally, alongside the colored contours, small closed contours or differently colored points are observed, indicating the presence of small holes due to collapsed gas bubbles or peaks formed by localized material solidification.

**Figure 10:**
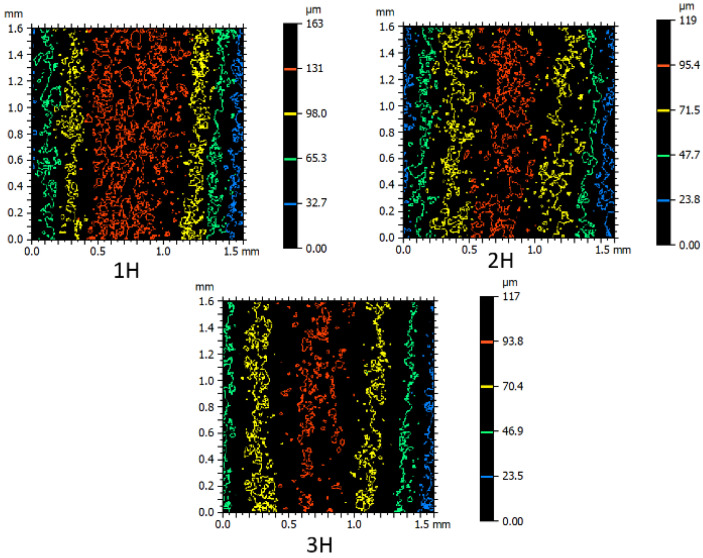
Contour color spectrometry to compare the cut samples with a medium-radius convex surface, produced through an electric discharge process by wire in three states: roughing, semi-finishing and finishing (Sample H, according to the nomenclature of Figure 2).

**Figure 11:**
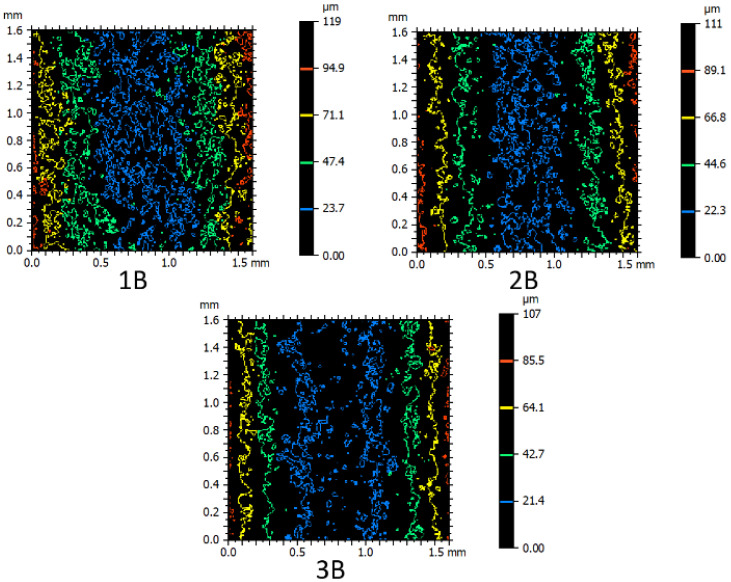
Contour color spectrometry for comparison of cut samples with concave surface with medium radius, produced through an electric discharge process with wire in three states: roughing, semi-finishing and finishing (Sample B, according to the nomenclature of Figure 2).

To provide a tangible reverse correlation between surfaces with convex and concave curvatures, Figure 10 and Figure 11 are presented, respectively. For comparison, samples with convex surfaces with average radii, produced through the wire electrical discharge process in rough, semi-finish, and finish conditions, are shown in Figure 10. Corresponding samples with concave curvatures are displayed in Figure 11. As the curvature radius increases, the number of closed contours appearing on the surface at different heights with different colors increases. In essence, the emphasis gradually shifts towards the difference in height at various points, influenced by the geometric features of the samples, as the height of peaks and valleys becomes more pronounced. This phenomenon is more evident on the recast layer of flat samples.

Figure 10 and Figure 11 exhibit similarities in the visual appearance of contours, regardless of the color of the contours, which indicates their elevation. In terms of the patterns and motifs present, the resemblance between Figure 10 and Figure 11 indicates the accuracy of executing nominal contours with specific radii, whether they are convex or concave. The black spaces between the colored contours, in the case of samples produced with different passes of the electrical discharge process, signify that the surface is more susceptible to deviations from the selected contour geometry to local defects and pits. Conversely, smaller black spaces between the colored contours indicate that the surface is more susceptible to defects resulting from the electrical discharge method than the selected contour geometry. Another difference between Figure 10 and Figure 11 is that each contour that appears red in samples with convex curvatures is blue in samples with concave curvatures. Similarly, green colors can be referred to as yellow, yellow as green, and red as blue in Figure 10 and Figure 11.

**Figure 12:**
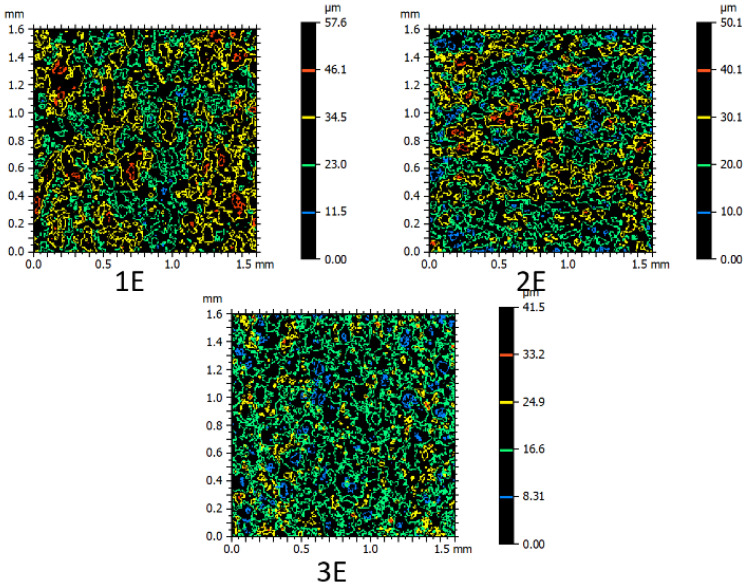
Contour color spectrometry for comparison of flat-cut samples, produced through wire electrical discharge process in three states: roughing, semi-finishing and finishing (Sample E, according to the nomenclature of Figure 2).

Similarly to the method used for comparing samples in previous figures, in Figure 12, a comparison was made for flat samples cut through one, two, and three passes. According to the figure, the closed contours in a sample cut through one pass were coarser and ranked first, followed by samples cut through two and three passes, respectively. In the sample cut through one pass, the largest closed contours are associated with yellow-colored contours, indicating surface irregularities that are intermediate in height between peaks and valleys. In this sample, the number of red-colored closed contours, representing the tallest peaks on the surface, is greater than in the other two flat samples. The blue-colored closed contours, indicative of the deepest valleys on the surface, are also fewer than in the other two samples.

None of the samples revealed a specific orientation for the contours in terms of distribution, but in the sample produced with one pass, the orientation of the closed contours appears to follow the path of wire movement. The sample produced with two passes is in an intermediate position in terms of the color and geometry of the contours. In the sample produced with three passes, the predominance of blue and red closed contours is contrary to that of the sample produced with one pass. By examining the longitudinal and transverse bounding axes of the imaging range and counting the intersection points of closed contours, it can be observed that the number of closed contours in the sample produced with three passes is higher than in the two- and one-pass samples, undoubtedly due to the implementation of electrical discharge with lower energy density.

**Figure 13:**
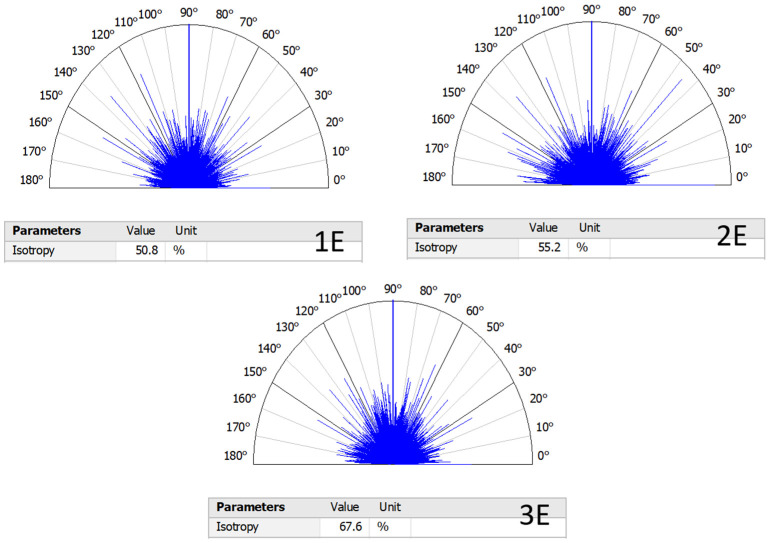
Comparison of surface profile anisotropy of flat-cut samples, produced through wire electrical discharge process in three states: roughing, semi-finishing, and finishing (Sample E, according to the nomenclature of Figure 2).

Utilizing the confocal laser scanning method to measure changes in the differences in peak and valley sizes across various orientations, another capability of the method has been leveraged. This method essentially reveals surface homogeneity. As shown in Figure 13, different angles around one of the central points of the surface, considered to be the focal point, are defined, and differences in peaks and valleys are examined along each angle. In many surface textures related to machining processes, complete heterogeneity is usually observed. However, considering the explanations provided earlier regarding the negligible difference between the direction of wire movement and the direction of advancement along the cutting path, this measurement method can be applied to the surface texture resulting from the electrical discharge process.

With the utilization of laser in-surface imaging, the homogeneity measurement method operates in a completely non-contact manner and is operation is based on the information obtained via confocal laser scanning. According to Figure 13, the homogeneity level in the recast layer corresponding to the sample cut through three passes is higher than the other two samples. In this comparison, the sample cut through two passes ranks second, and the sample cut through one pass ranks third. The relationship between the energy density applied in the single-pass electrical discharge and the roughness obtained on the recast layer surface is clearly evident in Figure 13. The homogeneity of the recast layer surface of the sample cut through two passes is eight percent higher than that of the sample cut through one pass. Regarding the sample cut through three passes, this value increased by 33 percent compared to the second-pass cut. Therefore, compared to the second pass, the third pass has a greater effect on the homogeneity of the surface.

**Figure 14:**
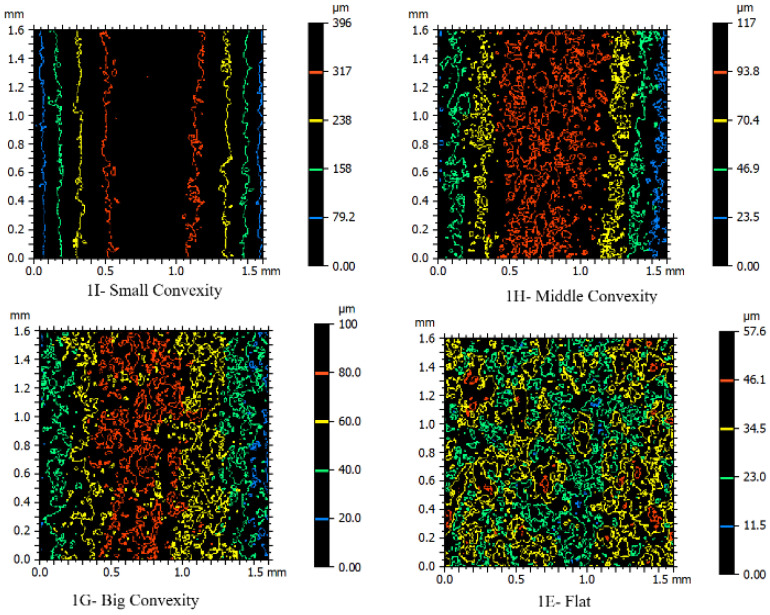
Contour color spectrometry for comparison of flat and convex-curved cut samples with small, medium, and large radii produced through electrical discharge process by wire in roughing condition or one pass (samples I, H, G, and E, according to the nomenclature of Figure 2).

To evaluate the contour shapes present on the surfaces in Figure 14, samples with different geometric features were considered together. It was previously explained that the contour shape is a function of surface geometric features, and their colors are a function of height from the origin point. Here, flat samples with convex curvatures of large, medium, and small radii were compared. Due to the color spectrum defined based on the minimum and maximum heights, each of the four samples exhibits red, yellow, green, and blue colors for their contours. If the basis for color changes in the color spectrum was adjusted based on the smallest convex radius (piece C), only one or at most two colors would be observed in the other samples. In the sample with the smallest convex radius (piece I), the contours are completely linear, and continuity between their start and end points is evident. As the convex radius increases in the sample with the medium convex radius, the contour lines transform into ranges that are particularly color-separated and entirely distinguishable from each other. In the largest convex radius, interference within the colors has appeared, and the small closed contours have increased. This indicates the closer effect of the prominences and depressions resulting from the recast layer surface to the geometric features of the contour. Ultimately, in the flat sample, it is not possible to imagine a clear boundary between the colored contours in terms of positional location. The number of small closed contours has also increased significantly in this case. In this situation, adherence to the properties of the recast layer surface over the geometric properties of the contour form is preferred.

## 4. Evaluation of Surface Roughness Resulting from Cutting with the WEDM Process on Different Contour Form Geometries

**Figure 15:**
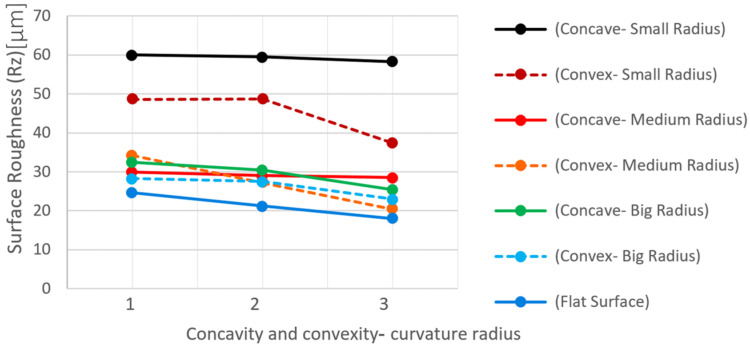
The diagram of the influence of the radius of curvature on the surface roughness with the Rz criterion (for samples with different geometric profiles and three electrical discharge methods in terms of the number of cutting passes).

A comparison of the roughness values obtained with the R_z_ criterion for samples produced with seven different geometric features for the contour form and three electrical discharge machining methods in terms of the number of cutting passes yielded the graph shown in Figure 15. The Rz parameter was chosen because it provides a more representative measure of peak-to-valley variations, making it more suitable for assessing the influence of different geometries and machining passes on surface quality. Unlike Ra, which represents an average roughness and may overlook localized surface irregularities, Rz is more sensitive to topographical changes. As observed, the roughness values decreased from samples produced with one pass (~65 µm for concave—small radius) to those produced with three passes (~62 µm for concave—small radius). The roughness measurements were carried out using the roughness-, profile-, and twist-measuring device Hommel-Etamic T 8000 and the form-measuring device Hommel Etamic F50, both manufactured by Jenoptik, which is located in Villingen-Schwenningen, Germany. This descending trend indicates the influence of energy density and adjustment parameters between the first to third passes on the height of surface prominences of the recast layer. The dispersion of roughness was lower in samples with less convex curvature compared to those with concave curvature. The flat sample had the lowest surface roughness with regard to all three methods, with one, two, and three passes (~22 µm, ~20 µm, and ~18 µm, respectively). In samples with convex curvature, the surface roughness increased as the convex radius decreased (e.g., ~50 µm for convex—small radius and ~30 µm for convex—big radius at the first pass). This trend is the same for a sample with concave curvature (e.g., ~40 µm for concave—medium radius and ~28 µm for concave—big radius at the first pass). The difference in roughness between samples with medium and large curvature radii was much less (~2–3 µm) than the difference between samples with medium and small curvature radii (~10–15 µm). A comparison of the roughness values with the Ra criterion also shows a similar trend, with minor differences from the previous method.

**Figure 16:**
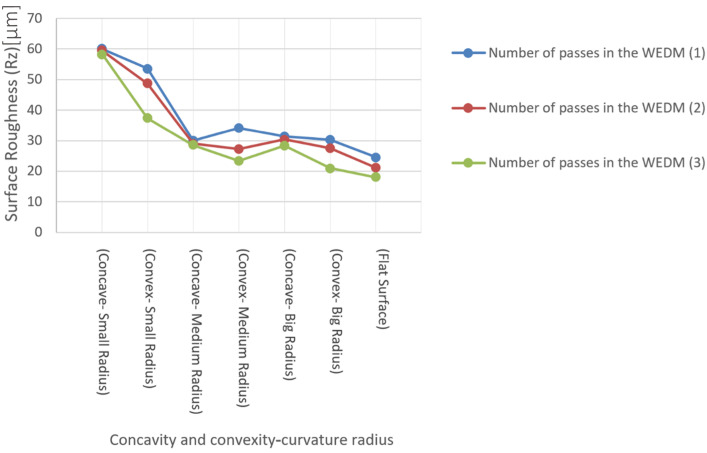
The graph of the influence of the radius of curvature of the samples on the surface roughness with the Rz criterion according to the number of passes in the WEDM process.

Approaching the recent test results from a different perspective, notable findings emerge. In Figure 16, instead of representing the number of cutting passes, the horizontal axis is dedicated to the seven geometric characteristics of the samples. As can be seen, points are considered for flat samples, those with convex curvature (with large, medium, and small convex radii), and those with concave curvature (with large, medium, and small concave radii). Finally, the trend of changes in roughness of the recast layer resulting from cutting is depicted in three different colors.

According to Figure 16, the roughness resulting from the Rz criterion with the relevant settings on the roughness tester, which included the selection of appropriate filtering techniques, the measurement length, repeatability factor, and the feed rate and traverse speed, from a single-pass cut is higher (~60 µm for concave—small radius) compared to two (~55 µm) and three passes (~50 µm). The difference in roughness between samples with convex and concave curvature, which have the smallest radius of curvature (convex—small radius and concave—small radius: respectively, pieces I and pieces C), is greater (roughness difference ~10–15 µm). This observation can be crucial when convex and concave curvatures coexist simultaneously in two areas of a contour form with slight curvature radii considered. In such cases, employing an electrical discharge machining method in terms of the number of passes cannot achieve uniformity in the recast layer’s surface in these two areas. Achieving this goal may require increasing the number of cutting passes only in the area with concave curvature.

**Figure 17:**
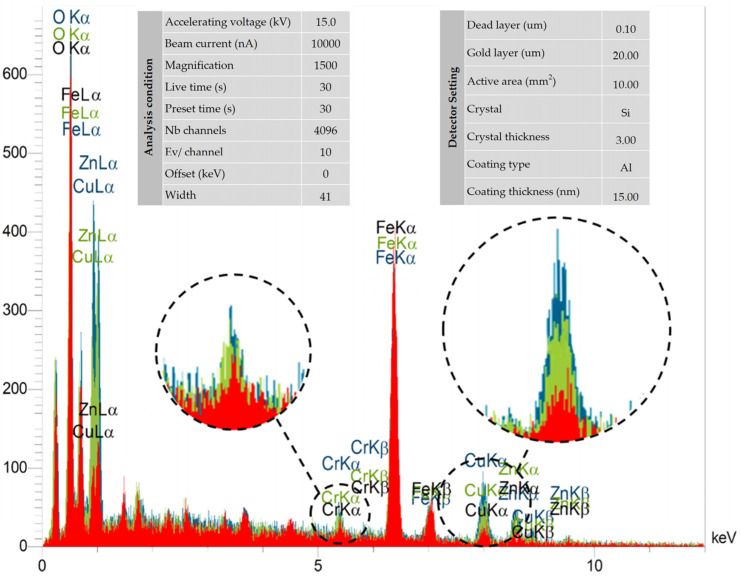
Adjustment factors and diagram of EDS spectrometry method, for the materials used to manufacture form-cutting tools.

An example of the EDS spectra, measured for the material of the cutting tool (HSS), is depicted in Figure 17. The analysis settings and the detector parameters, along with the results of this method, presented in numerical form, are also illustrated in Figure 17. Copper and zinc elements are considered to be the predominant elements in the material of the wire used in the wire-cut machine to investigate the possibility of their retention on the recast layer’s surface.

Regardless of the geometry of the contour form, it is important to note initially that, in the comparisons made between the output spectra analysis of EDS, consistent observations were obtained for all geometric characteristics based on the number of cutting passes in the electrical discharge machining process. Based on this, when the three spectra obtained using the EDS method matched each other, differences were observed in the percentage of selected elements on the recast layer’s surface.

As depicted in Figure 17, three different colors are used to represent the EDS spectra. The red, green, and blue colors correspond to the EDS spectra of recast layers with three, two, and one pass in the wire electrical discharge machining process, respectively. Two elements, copper (Cu) and zinc (Zn), were used to investigate the effects of wire-cut residuals on the recast layer’s surface. The intensity of the presence of these two elements on the recast layer varies from high to low for one, two, and three wire-cut passes, respectively. This implies that in the second and third passes, where the electrical discharge energy is lower, the erosion of the wire has been such that its effects on the recast layer’s surface are less noticeable.

As can be seen, the intensity of the presence of various elements is expressed on the vertical axis, based on an “arbitrary unit”. Therefore, utilizing this method ensures that the measurement remains invariant to scale variations or changes in the unit of measurement.

This analysis is crucial because it helps us to better understand how the energy density and flushing effects influence the distribution and presence of specific elements (copper and zinc) on the surface of the samples. By investigating the intensity of copper and zinc presence in the upper and lower regions, we can assess the impact of wire erosion, element migration, and heat transfer during the electrical discharge process. This knowledge is vital for optimizing the EDM process parameters to improve surface quality, enhance material properties, and control the recast layer formation. These findings are particularly important when designing tools or parts with specific geometries, as they provide insights into how different machining passes affect the final surface composition.

**Figure 18:**
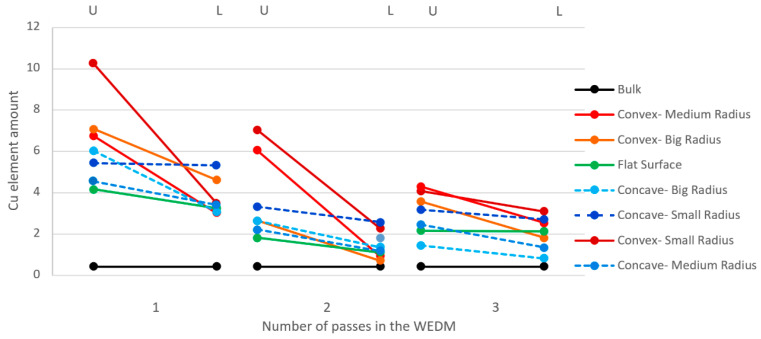
The intensity of the presence of copper (Cu%) in the upper and lower regions of the cut samples (with different contour geometries) through the process of discharging electricity via wire.

With this background in mind, the intensity of the presence of the copper element is depicted in Figure 18. It is evident that the data scattering from the single-pass (~10.5% for convex—medium radius) to the triple- pass (~3% for convex—medium radius) production method follows a decreasing trend. Additionally, the intensity of the presence of copper in the upper region of the samples is higher compared to that in the lower region (e.g., for concave—small radius, Cu% in the upper region decreases from ~9% to ~2%, while in the lower region, it decreases from ~7% to ~1.5%). This could be due to the 100% presence of copper in all peripheral areas of the wire during its entry into the sample, as despite the damages incurred during the pass-through of the samples, the surfaces contain layers that have been oxidized with carbon, to some extent, adhered elements from the workpiece material among the copper and zinc elements. This might explain the decrease in the presence of copper in the lower region of the workpiece. Assuming equal amounts of dielectric fluid flushing occur in the upper and lower regions of the samples, another reason could be the wire-feeding direction, which always descends from top to bottom and can itself serve as a factor for removing the formed elements during electrical discharge. The difference in the intensity of the presence of copper between the upper and lower regions is more pronounced in samples with concave curvature (~3% difference for concave—medium radius). In both curvature directions (concave and convex), as the curvature radius decreases, the intensity of the presence of copper increases (for convex—small radius, cu% starts at ~11% for one pass and decreases to ~3% for three passes). The sample with a flat surface contained a minimal amount of copper following both two (~2.5%) and three (~1.5%) cutting passes.

**Figure 19:**
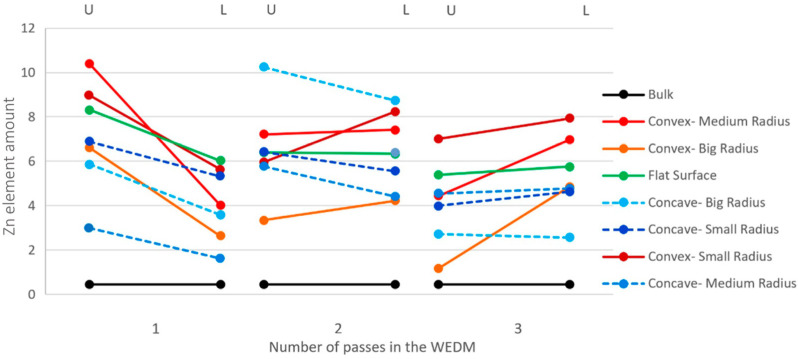
The intensity of the presence of zinc (Zn%) in the upper and lower regions of the cut samples (with different contour geometries) through the process of discharging electricity via wire.

Similarly to the analysis of copper intensity, an investigation into the presence of zinc has been conducted. It is evident from Figure 19 that the trend of presence intensity between the upper and lower points of the sample is descending for single-pass cutting (Zn% ~10% for convex—medium radius) and ascending for three-pass cutting (Zn% ~4% for convex—medium radius). This indicates that in electrical discharge machining (EDM) with higher energy density, the presence of zinc (compared to the upper region) is less intense in the lower region of the sample, but at lower energy densities, it is higher in the same region (e.g., for concave—small radius, the Zn% in the lower region increases from ~6% to ~9%)

The first point to note in this regard is that in EDM with higher energy density, the level of wire erosion is higher, and the surface where an increased amount of zinc is observed is generally higher. Considering the low melting point of zinc, it is among the last elements to undergo localized melting and solidification after the removal of local heat. However, due to the heat generated from adjacent points where electrical discharge occurs with a time delay along the cutting path, it melts earlier than other elements and is washed away during the flushing action. Therefore, at lower energy densities, its crystallization in the lower regions of the workpiece is less affected by new sparks resulting from the wire penetration into adjacent points. This stability leads to the entrapment and persistence of the zinc element in these regions.

In reality, a threshold of energy density can be envisioned where, considering the generated heat, the workpiece’s heat-transfer coefficient, and the intensity of dielectric fluid flushing, the presence intensity of zinc is minimal (Zn%~1% for a flat surface after three passes). At higher energy densities, due to significant wire erosion, and at lower energy densities, due to reduced susceptibility to electrical discharge influence in adjacent points, zinc is more prevalent.

A comparative analysis was conducted, in which two samples of high-speed steel (HSS) with different thicknesses were used to assess the variations in the recast layer characteristics between the upper and lower regions of the samples, or in other words, the entry and exit positions of the wire in wire electrical discharge machining (WEDM). One sample with dimensions equivalent to the previous seven samples (considered for the geometric contour effect analysis) was chosen as the thin thickness, and another sample with five times the thickness was used for comparison.

**Figure 20:**
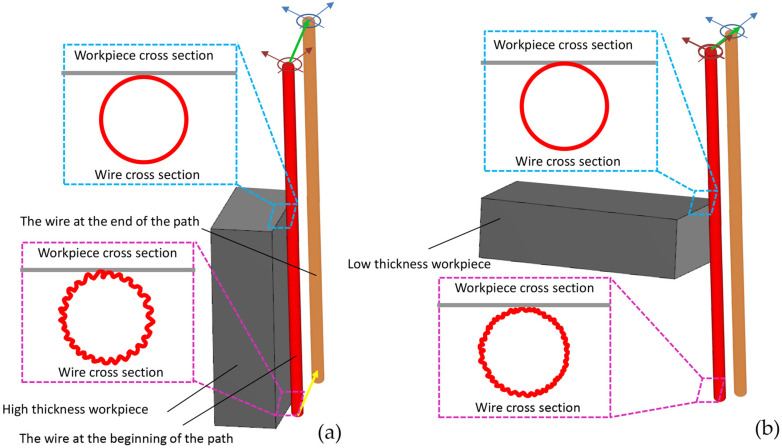
The length of the contact segment between the wire and the workpiece surface in (**a**) high- and (**b**) low-thickness workpieces.

As depicted in Figure 20, due to the flatness of the sample surface and the direct cutting path, the engagement length of the wire with both samples is equal. However, considering the fivefold thickness in one of the samples, undoubtedly, the wire erosion rate is higher due to the longer length of contact with the workpiece. Therefore, its effect on the difference between the upper and lower regions of the sample should be manifested.

**Figure 21:**
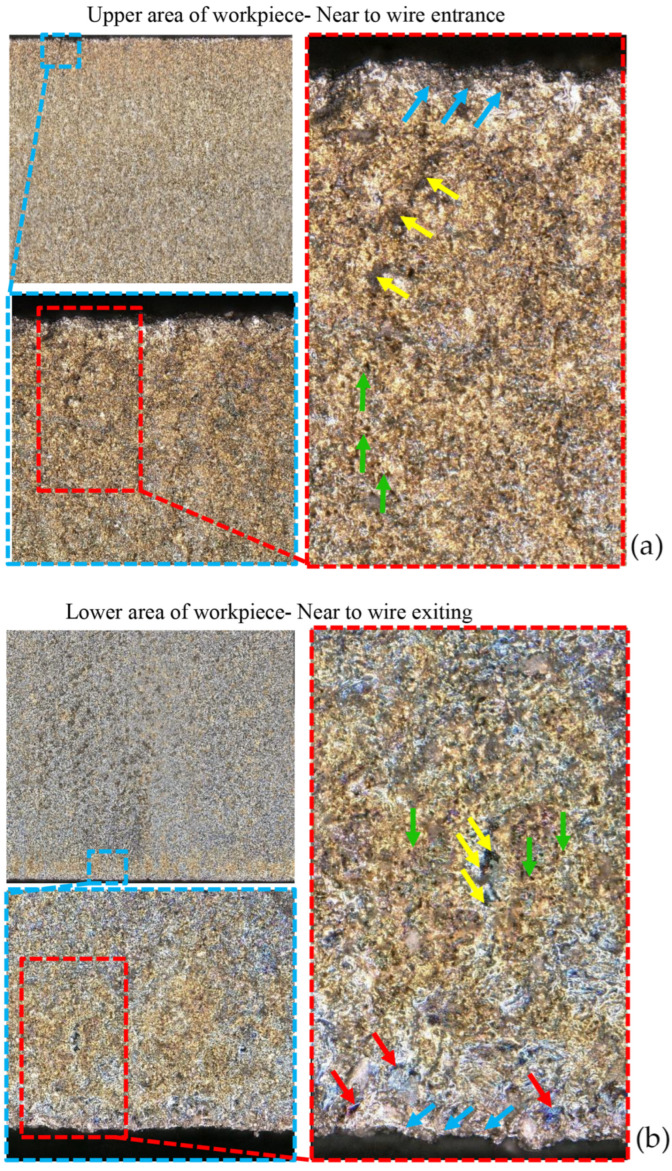
The difference between the recast layers on a sample with a high thickness, after the WEDM process in the (**a**) entry and (**b**) exit areas of the wire.

According to Figure 21, the differences in the surface characteristics in the upper and lower regions of thicker-cast layer samples are more pronounced. The first noticeable difference is the presence of different background colors in these two regions. In the lower region, closer to the wire exit point, color changes are observed, which may be due to the accumulation of separated elements resulting from wire erosion during the electrical discharge machining process. These color changes increase as the lower edge of the sample is approached.

In Figure 21, the areas highlighted with blue arrows indicate the ups and downs created at the upper and lower edges of the sample, which are sometimes used as cutting edges in milling operations. Certainly, achieving a smooth and straight edge can significantly impact the cutting tool’s lifespan. The green arrows represent traces of gas bubbles formed during the melting of the material due to the energy density resulting from electrical discharge and dissipated during solidification. The yellow arrows, indicating the presence of small dark particles among the highs and lows of the recast layer’s surface, suggest the deposition of carbon or other alloying elements present in the process.

Finally, the red arrows, which are only present in the lower region of the thicker samples, represent colorful spectra that appear to have frozen superficially after the underlying layer’s solidification. It is possible that these colors are the result of the washout effect of the upper regions and neighboring areas with a time delay dependent on the advancement rate of the electrical discharge process. Alternatively, they may result from the rapid solidification of elements with a high melting point present in the wire EDM, which may have occurred due to erosion caused by electrical discharge in other points after passing through the previous point.

## 5. The Surface Properties of the Recast Layers

**Figure 22:**
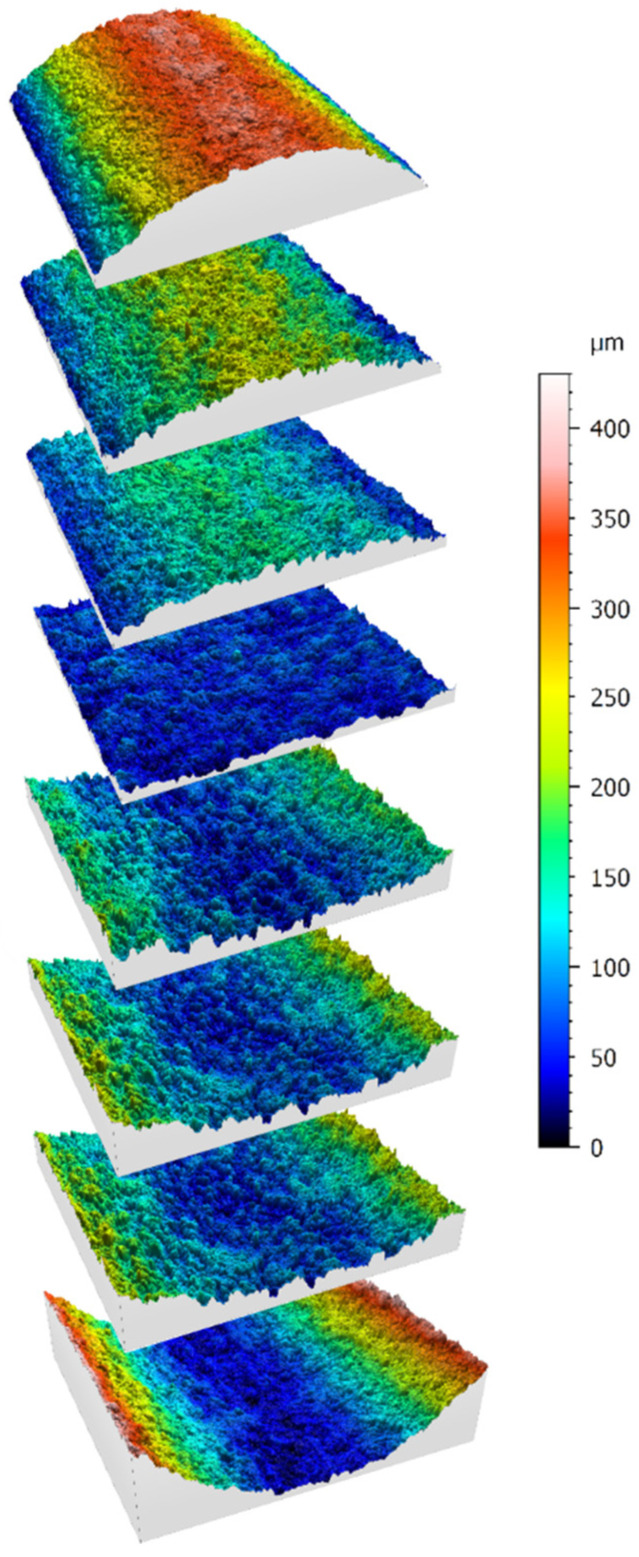
Comparison of color spectrum from CLSM 3D surface profile of different geometric samples created by the WEDM process.

For the investigation of the surface properties of the recast layers present on the cut surfaces of the samples, the confocal laser scanning microscopy method was employed. Given the advantages of this method in creating three-dimensional images, many surface features could be effectively measured and compared among the available samples. Figure 22 depicts cross-sectional views of seven samples with various geometries concerning contour forms, which are created in three dimensions and using color spectra. This image pertains to samples created through three passes in wire electrical discharge machining.

The square sections that were captured had dimensions of 2.2 mm by 2.2 mm (0.0866 inches by 0.0866 inches) to prevent any measurement errors resulting from graphic matching or alignment motions in the longitudinal and transverse axes of the microscope. The use of a flat mode was avoided. As previously mentioned, the middle area of the samples was used for imaging to conceal the differences between their input and output regions.

**Figure 23:**
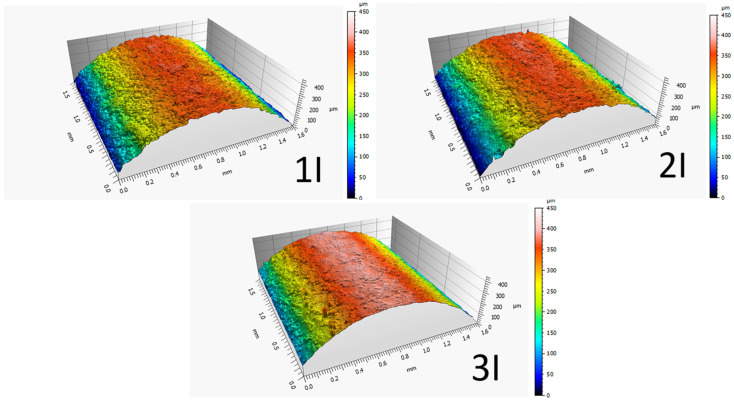
Comparison of the effect of different cutting conditions (one, two, and three passes) on the creation of the recast layer according to a three-dimensional scan of the contour of the form (Sample I, according to the nomenclature of Figure 2).

In Figure 23, samples produced by one, two, and three passes in the electrical discharge machining process were compared. Geometrically, these samples had concavities with an average radius among the radii of curvature delineated. As the color spectra depict, the sample produced with a single pass exhibited larger peaks and valleys on the surface of the recast layer due to the higher energy density used in the electrical discharge process.

The peaks present on the sample produced with a single pass in the electrical discharge machining process, as depicted in Figure 23, clearly indicate the reason that more wear is observed on the form-cutting tools produced using a similar method, which is their mechanical contact with the machined surface of the workpiece [34]. Indeed, it can be said that these peaks gradually decrease in height due to continuous engagement with the workpiece, one after another, and by removing them, other peaks become new frictional elements interacting with the surface of the workpiece. This process continues until, according to established definitions of tool life, it is no longer feasible to utilize the tool further.

In the samples depicted, the wire travels in a direction parallel to the curvature axis, as is clearly evident in the images in Figure 23. However, no specific orientation is observed in the defects and cavities present on the surface. Therefore, when constructing cutting tools, the choice of the wire passage direction can only be based on the geometric constraints of the contour form.

## 6. Data Related to the Recast Layer in the External and Internal Corners of the Form-Cutting Tools

Some points on the contour form have been considered critical points to allow for a more precise comparison in terms of their behavior under different passes in the EDM process. Both internal and external corners are among these critical points. In the seven samples created to investigate the effect of contour form geometry on the features of the recast layer, both types of corners were present. Internal corners were characterized by a convex contact surface and a flat surface, while external corners had a concave contact surface and a flat surface.

**Figure 24:**
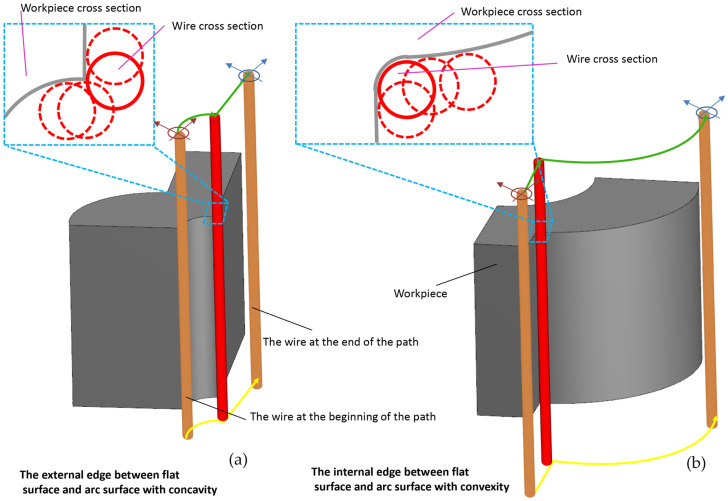
The length of the contact segment between the wire and the surface of the workpiece in the (**a**) external and (**b**) internal corners.

As shown in Figure 24, the length of the wire–workpiece contact area differs in external and internal corners. In external corners, based on the geometric location of the wire center along the programmed path, the contact points on the wire periphery move from the coordinates where the external corner is located to new contact points on the wire periphery along a different direction. Conversely, in internal corners, due to the change in wire direction, there is a dwell time in the contact area between the points on the wire periphery and the workpiece. Therefore, it is likely that the sharpness of external corners is reduced because, compared to normal points on the surface, the external corner is subjected to the energy of the EDM environment from both sides and in two stages. In internal corners, there is also a greater likelihood of increasing the corner radius because the dwell time of the wire causes the coordinates related to the internal corner to be exposed to the EDM environment for a longer period.

**Figure 25:**
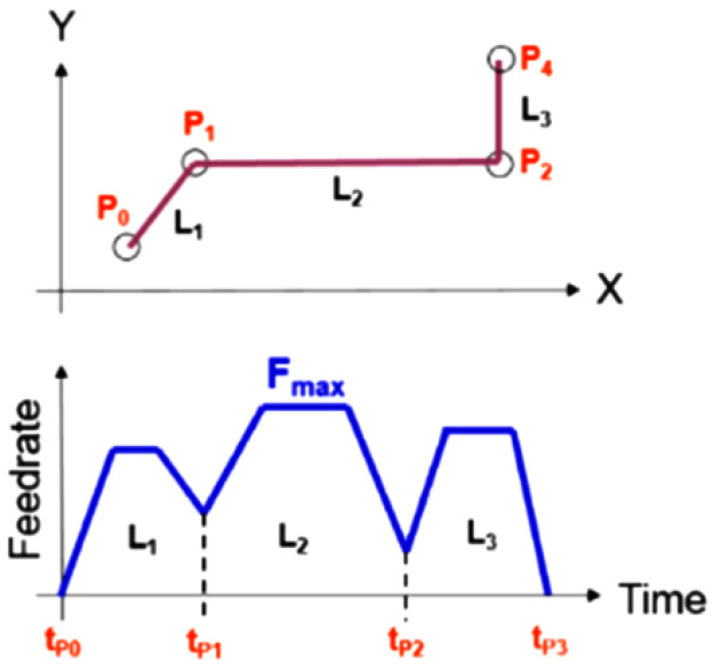
The process of the changes in the feed rate in three consecutive lines from a numerical control program [35].

A displacement diagram showing three continuous tool paths from a numerical control program, along with the trend of speed changes, especially at the end points of one path and the beginning of the next, is depicted in Figure 25. Although a predetermined value for feed rate is considered in the numerical control program, there is always a linear trend for increasing the feed rate from the starting point of motion on one or more axes to reach the defined nominal value. In fact, at each change in direction or at the start of each path in the program, the feed rate starts from zero and reaches the maximum defined value. Then, before completing the path near the endpoint, it is reduced again to zero. With this interpretation, it is clear that the feed rate is low before and after the point where two continuous paths in the program terminate and begin. This can be considered a challenge, especially in the internal corners, in the EDM process.

**Figure 26:**
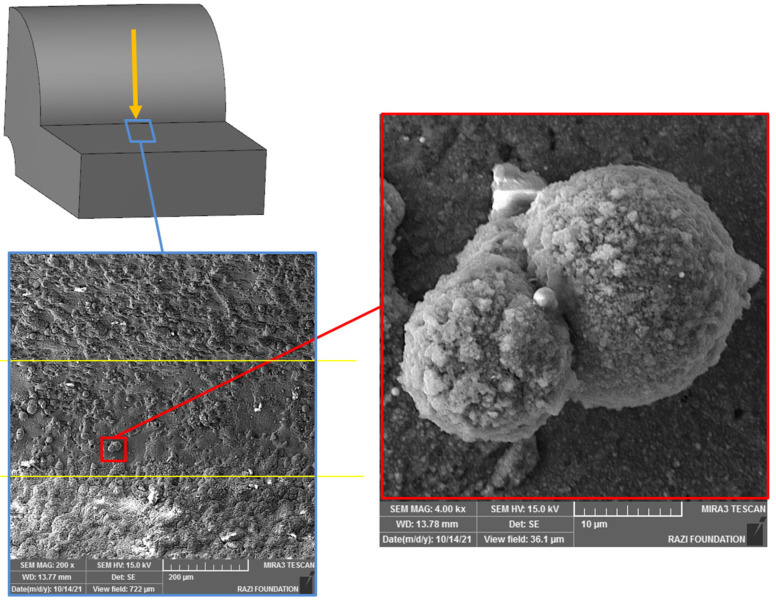
The indentation area (corner) between the flat surface and the convex curvature of the tool produced with three wire-cut passes (the surface between the curvature of the upper area and the flat surface).

**Figure 27:**
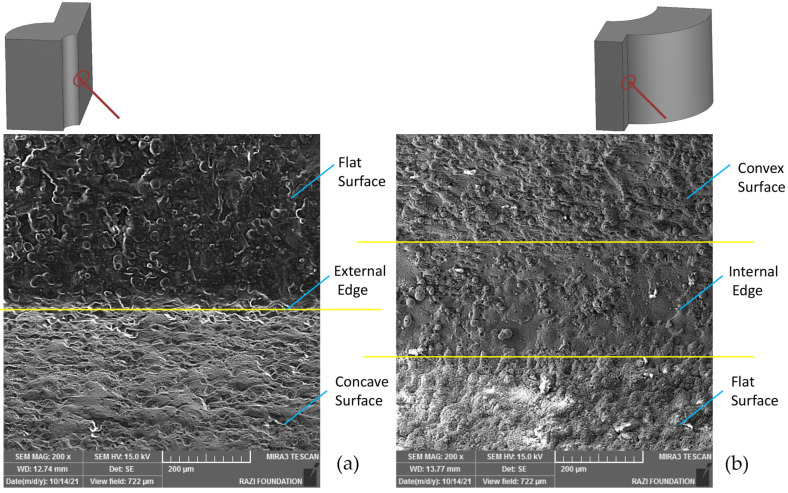
The difference between the recast layers created under the same WEDM conditions but which differs in terms of the features on the workpiece (here, the cutting tool—(**a**) external and (**b**) internal corners).

Figure 26 depicts the presence of a very small spherical mass captured by an electron microscope. The imaging location is the inner corner of one of the samples where the merging of surfaces with convex curvature and flat surfaces has occurred. The existence of this spherical mass indicates the aforementioned interpretation of the presence of dwell time in the inner corners. In fact, the freezing time has been so high that the mass has moved towards a spherical shape. Such masses are not found in many of the recast layers observed on flat surfaces. Figure 27 addresses the differences between the recast layers present in the outer and inner corners. It is observed that there are differences in the visual properties of the recast layer before and after the inner and outer corners. Although these visual differences may be due to the angles that adjacent surfaces have with inner and outer corners, studying the surfaces of different samples with different geometric features using confocal laser scanning microscopy has shown that these differences exist.

The recast layer surface resulting from the wire electrical discharge machining process appears smoother on the inner corners. This may be due to the dwell time and the increased presence of wire in this area. Spherical masses are clearly visible in this area. In the outer corners, it is also observed that the sharpness of the outer corner has a direct correlation with the height of surface defects.

## 7. Conclusions

The main assumption for each type of profile form is based on a combination of four states: concave curvature, convex curvature, a flat surface, and an inclined surface. Accordingly, HSS test samples were fabricated as cutting tools with three different radii of convex curvature, three different radii of concave curvature, and a flat surface. During the WEDM operation, one pass was used for roughing, two passes for semi-finishing, and three passes for finishing. Additionally, the difference in surface layer quality between the upper workpiece area or wire entry point and the lower workpiece area or wire exit point was studied. Finally, the effect of direction, curvature magnitude, and number of passes in the WEDM process on the recast layer was demonstrated. It was observed that with an increase in the number of passes in WEDM, the thickness of the recast layer decreases, and the uniformity of the cutting contour in areas close to the wire entry point is greater than in areas close to the wire exit point. Surface roughness values decreased from samples produced with one pass to those produced with three passes. A descending trend relating to the effect of energy density and control parameters between the first and third passes on the height of surface recast layer protrusions can be observed. There is less scattered roughness in samples with convex curvature than in samples with concave curvature.

In convex samples, the surface roughness increases as the convex radius decreases. This trend is consistent for concave curvature samples as well. The difference in roughness between samples with average curvature and large curvature is much smaller than the difference between samples with average curvature and small curvature.

Regardless of the curvature radius, the scatter of roughness in concave curvatures is less than that in convex curvatures. Therefore, in contoured forms with curvature, different behaviors regarding the phenomenon of wear may be observed due to the differences in surface recast layer properties.

If the flat surface is also considered as a curvature with a very large radius, it is evident that the challenges associated with achieving uniform roughness in the recast layer increase with decreasing curvature radius.

The length of the contact area between the wire and the workpiece surface differs in the outer and inner corners. In the outer corners, based on the geometric location of the wire center along the program path, contact points on the wire surface pass through coordinates where the outer corner is located to provide new contact points on the wire surface in a different direction. Conversely, in inner corners, due to the change in the wire’s movement direction, there is a dwell time in the area of contact between points on the wire surface and the workpiece. As a result, the sharpness of the outer corners is reduced, and compared to points located on the surface, the outer corner is subjected to energy from the outer wire discharge in two stages. In inner corners, there is also an increase in the corner radius because the dwell time of the wire causes the coordinates related to the inner corner to be exposed to the energy of the outer wire discharge for a longer period.

## Figures and Tables

**Table 1:** Chemical composition and properties of the HSS materials used [32].

BÖHLER Grade	Conventional High-Speed Steel								
	Chemical Composition in %							Standards	
BÖHLER S200	C	Cr	W	Mo	V	Co	Others	DIN/EN	AISI
	0.76	4.1	18	-	1.1	-	-	<1.3355> HS18-0-1	T1

## Data Availability

No datasets were generated or analyzed during the current study.

## Glossary

CAD	Computer-Aided Design.
CAM	Computer-Aided Manufacturing.
CLSM	Confocal Laser Scanning Microscopy.
EDM	Electro-Discharge Machining.
EDS	Energy-Dispersive X-Ray Spectroscopy.
FA	Actual Feed Rate of Electrode in Cutting Direction (Base of the Mitsubishi Table).
H	Distance Between Electrode Center to Workpiece Surface (Base of the Mitsubishi Table).
HSS	High-Speed Steel.
IP	Intensity of Power (Base of the Mitsubishi Table).
OFF	Pulse Interval Time (Base of the Mitsubishi Table).
ON time	Pulse Time During Discharging (Base of the Mitsubishi Table).
rpm	Revolutions Per Minute.
SEM	Scanning Electron Microscope.
VG	Gap Voltage During Discharging (Base of the Mitsubishi Table).
WEDM	Wire electrical discharge machining.
WS	Wire Traveling Speed (Base of the Mitsubishi Table).
WT	Wire Tension (Base of the Mitsubishi Table).
DF	Dielectric Fluid Flow Rate (L/m) (Base of the Mitsubishi Table)
